# Competitive Psychological Disposition and Perception of Performance in Young Female Soccer Players

**DOI:** 10.3389/fpsyg.2019.01168

**Published:** 2019-05-22

**Authors:** Aurelio Olmedilla, Roberto Ruiz-Barquín, Francisco Javier Ponseti, Francisco Javier Robles-Palazón, Alexandre García-Mas

**Affiliations:** ^1^Department of Personality, Evaluation and Psychological Treatment, University of Murcia, Murcia, Spain; ^2^Department of Developmental and Educational Psychology, Autonomous University of Madrid, Madrid, Spain; ^3^Department of Pedagogy, University of the Balearic Islands, Mallorca, Spain; ^4^Department of Physical Activity and Sport, University of Murcia, Murcia, Spain; ^5^Department of Psychology, University of the Balearic Islands, Mallorca, Spain

**Keywords:** psychological profile, performance perception, mental skills, football, women

## Abstract

The athletes’ psychological disposition is a factor that is increasingly considered by researchers as a key to sports performance, even as a mediator between the physical, technical and tactical abilities of the athlete and their competitive performance, thus acquiring great relevance in training and in sports performance. The purpose of this study is to analyze the psychological characteristics of young soccer players and their relation to their performance perception, made both by the player herself and by their coaches. The sample is composed of 108 women (*M* age = 15.53, *SD* age = 1.05), with ages between 13 and 17 years (13 years, *n* = 1, 14 years, *n* = 18, 15 years, *n* = 36, 16 years, *n* = 29, 17 years, *n* = 24), and with a sport practice experience of 7.27 years on average (*SD* = 2.64). For to address this aim, we used the Psychological Characteristics related to the Sport Performance Questionnaire (CPRD) and the Psychological Skills Inventory for Sports (PSIS). In addition, regarding the evaluation of performance perception, an *ad hoc* short questionnaire was created, composed by one question addressed to the player and one directed to the coach. The results indicate that the group of players of the under-16 category obtained higher scores in all the psychological dimensions than the U-18 players, showing significant differences in Team Cohesion (*p* < 0.048). Regarding the degree of congruence between the player’s psychological features, and the player’s and coach’s performance perceptions, the results show statistically significant and negative correlations between the Team Cohesion factor and the athlete’s own outcome perception for the match #1 (rxy = -0.479; *p* < 0.001), and match #2 (rxy = -0.402; *p* < 0.01). The results of this study may contribute to establish the differences between different constellations of psychological characteristics according to the categories of competition and their relationship with the perception of performance. This knowledge can be used by sports professionals: coaches, psychologists, physical educators, etc., in order to help athletes to reach their maximum performance.

## Introduction

Soccer is a high-intensity intermittent team sport that requires technical, tactical and physical skills (Stolen et al., 2005), as well as psychological (Gledhill et al., 2017; Olmedilla et al., 2018b) for optimal performance. Soccer is one of the most researched sports in the scientific literature (Reilly et al., 2000; Datson et al., 2014). However, most of studies have been conducted traditionally with male soccer player samples, and only recent studies have analyzed female soccer player cohorts (Vescovi and McGuigan, 2008). Nowadays, female soccer has a great participation in United States (Markovits and Hellerman, 2003) and begins to be considered an attractive sport by women athletes in Europe and other countries around the world (Wright, 2014). Therefore, a greater research about the main factors related to the female soccer players’ performance is clearly required.

Starting from the theoretical model of Gagné (2004) (Differentiated Model of Gift and Talent, GMGT) to work simultaneously with personality traits, acquired skills and psychology requirements for the sports competition, it has been considered that the psychological disposition is one of the factors that have been strongly added to this analysis (Olmedilla and Dominguez-Igual, 2016; González, 2017). Considered as intermediary between players’ physical, technical and tactical abilities (Morris, 2000; Castilla and Ramos, 2012; Abdullah et al., 2016; Swann et al., 2017), psychological disposition is one of the keys for the sport performance due to the influence that it would have in the player’s competitive success (Anderson et al., 2014; Arthur et al., 2017). In this sense, some studies have found that physiological variables represent between 45 and 48% of the sport performance, and when the psychological variables were added, the proportion of variance explained increased around 79–85% in certain sports like wrestling (Nagle et al., 1975; Silva et al., 1981; Bali, 2015; Abdullah et al., 2016; Olmedilla et al., 2017a).

One of the approaches for studying the role of the psychological variables in sport performance is based on the personality traits (Mahoney and Avener, 1977; Gould et al., 1981; Burnik et al., 2005; Rasmus and Kocur, 2006; Cabrita et al., 2014), as the recent published study by Danielsen et al. (2017) where a relationship was found between the influence of mental toughness and the level of competition in female soccer players. Another approach studies differences between athletes and non-athlete population. For instance, Malinauskas et al. (2014) found higher scores in Consciousness in athletes than in non-athlete population, and greater Extraversion in team sportspeople in comparison with athletes from individual sports. Steca et al. (2018) found that athletes with better success in their careers revealed higher punctuations in all the Big-Five personality dimensions.

Given that these results do not show clearly a specific personality profile to distinguish between athletes and non-athlete population (Weinberg and Gould, 2014), neither result really useful for the sport performance, researchers have focused on the study of the relationship between athletes’ mental strategies, abilities and behaviors, and their subsequent performance (Romero et al., 2010; Álvarez et al., 2014; García-Naveira et al., 2015). This approach has been able to provide a better knowledge of the most relevant psychological characteristics for athletes, as well as to explain the differences between diverse sport participants and/or tactical positions in the same sport (Olmedilla et al., 2015). Also as they indicate Baillie et al. (2014, p. 406) “personality may also play a central role in determining the goodness of the fit between an athlete or team and a (psychologist) practitioner,” it could serve to distinguish male soccer players from female soccer players, and thus establish working hypotheses about the most appropriated psychological intervention to assist the sport performance; the knowledge of an athlete’s psychological profile enables a better understanding, the improvement of the communication procedures with himself and the increase in training effectivity (Martínez-Ferreiro, 2016), and it will result in better mental preparation and better psychological skills acquisition related to the sport success (Bahrololoum et al., 2012; Olmedilla et al., 2018c).

In recent years the number of works that show an especial attention in the study of the relationship between psychological factors and sport performance in female soccer has increased considerably (Danielsen et al., 2017). Specifically, the main psychological factors studied have been self-esteem and anxiety control (Williams, 2017), the prediction of the goal orientations suggesting coach’s specific actions (Granero-Gallegos et al., 2015; Domínguez-Escribano et al., 2017), some mental health indicators as depression and anxiety (Junge and Feddermann-Demont, 2016; Olmedilla et al., 2018a), and mood states (Arroyo del Bosque et al., 2016). Thus, it seems interesting the suggestion made by Rutkowska and Bergier (2015), who emphasized the importance of examining the women soccer players’ psychological profile due to the relevant information derived from this evaluation for improving training programs, primarily in mental training and in soccer players close to the professional level of performance (Martín-García, 2003).

In summary, sport performance is determined by a physical, technical and tactical systematic practice, necessary to achieve an elite level in soccer (Haugaasen et al., 2014). The same practice should be applied to the psychological training, strengthening the psychological resources for the sport competition; in this sense, the review published by Gledhill et al. (2017) stated 48 social and psychological factors linked to the talent development in soccer (e.g., adaptive lifestyle choices, practice and play behaviors). Similarly, there is a clear need to use women soccer players given the lack of studies that analyze this population in comparison with their male counterparts. Therefore, the main purposes of the current study were: (1) to describe the psychological characteristics of a Spanish sample of female young soccer players, and (2) to determine the differences between under-16 and under-18 age-groups, as well as their relation and congruence with perception of performance by the soccer player herself and her coach.

## Materials and Methods

### Participants

The sample was formed by 108 players that participated in the National Championship of Autonomic Selections of Spain of female soccer, in the categories under-16 and under-18. Mean age was 15.53 ± 1.05 years and mean of years practicing the sport was 7.27 ± 2.64 years, and 1.75 ± 0.92 years of experience in the highest category. The female players train an average of almost 3 sessions a week (2.98), lasting between 1 and 3 h each session, 102.64 ± 17.42 min on average and a total weekly training time of 304.44 ± 74.26 min on average.

### Measures

Psychological variables were assessed using the Psychological Characteristics Related to Sport Performance Questionnaire (CPRD, Gimeno et al., 2001), based on the Psychological Skills Inventory for Sports (PSIS, Mahoney et al., 1987; Mahoney, 1989). The questionnaire consists of 55 items graded in a 5-option Likert scale (from totally disagree to totally agree). It also includes a response option “I do not understand” to avoid missing answers.

Characteristics related to the Sport Performance Questionnaire includes five subscales: Stress Control (SC), Influence of Performance Evaluation (IPE), Motivation (M), Team Cohesion (TCOH), and Mental Skills (MSK), showing acceptable values of internal consistency for the total scale (α = 0.85) and for most of the subscales (αSC = 0.88; αIPE = 0.72; αM = 0.67; αTCOH = 0.78; αMSK = 0.34). According to the authors, the low internal consistency of MSK is probably related to it tapping a wide range of different skills but authors still keep the subscale due to the factorials loads showed by the items of this factor.

Stress Control consists of 20 items and refers athlete’s responses to potentially stressful situations and other training and competition demands. Higher scores denote athlete has management skills to cope with sport-related stress. IPE consists of 12 items and regards to athlete’s responses to situations in which he/she or people close to him/her judges his/her performance. It also includes an assessment about antecedents of athlete’s performance judgment. Higher scores mean the athlete can control the impact of performance judgment. M consists of eight items referring to basic motivation to sport performance and achievement, as well as to the regular training and competition activities. Higher scores indicate strong motivation and commitment to competitive sport practice. MSK consists of nine items and assess the use of different mental skills which are related to sport performance. Higher scores express better psychological resources to improve his/her performance. TCOH includes six items and assesses the extension the athlete feels attracted and identified with the sport group. This scale has not been used in this study due to the nature of the target sports.

To assess the perception of performance, an *ad hoc* instrument was created with a question addressed to the player, and a question addressed to the coach, and in both cases they had to answer on a liker scale of 0 (I played very badly) to 10 (I played very well).

### Procedure

After the corresponding author’s institution IRB approval (UM1551/2017), athletes were contacted by Football Federation of the Region of Murcia (FFRM) and football sport clubs. The researchers explained to both parents and athletes about the objectives and use of the information, and those who voluntarily participated signed an informed consent. In the cases in which the players were under 18 years of age, the signature of the parents was required. The data collection was made during the National Championship of Women’s Autonomous Soccer Teams held in Murcia (Spain) in December 2017. In the Championship participated the selections of Balearic Islands, Castilla la Mancha and the Region of Murcia in both categories. Authorization was requested to the Football Federation of the Region of Murcia (organizer of the event) and the coordinators of the different selections were informed about the objectives of the study. The CPRD questionnaire was administered in the accommodation hotel of the selections and during the leisure time between competitions. The evaluation of the perception of the performance was carried out right at the end of each game (from a total of two matches), asking first the players about their performance in the match, and then the coach of each team about the performance of each of their players in the match.

### Data Analysis

The analysis of data used have been: measures of tendency of central (means); dispersion measures (standard deviation); frequency analysis; normality tests using the Kolmogorov–Smirnov test; analysis of difference of means for two independent samples by the Mann–Whitney U; mean difference analysis for two correlational samples using the Wilcoxon W statistic; correlational analysis using Spearman’s correlation coefficient. Statistical analyzes were carried out using the statistical package SPSS 22.0.

## Results

Table 1 shows the descriptive analysis of the total sample of soccer players, and the application of the normality test (Kolmogorov–Smirnov) for the five factors, of which Team Cohesion (*p* < 0.001) and Motivation (*p* < 0.01) are not distributed normally.

**Table 1:** Descriptive values of the five factors of the CPRD questionnaire and application of the Kolmogorov–Smirnov normality test.

	*M*	*SD*	Minimum	Maximum.	Centil	K–S	Sig.
SC	51.26	11.16	24.00	80.00	65	0.076	0.155
IPE	25.83	6.70	11.00	44.00	55	0.080	0.085^†^
M	23.86	4.04	10.00	32.00	85	0.110	0.003^**^
MSK	21.37	4.09	11.00	33.00	55	0.073	0.200
TCOH	19.94	2.85	4.00	24.00	65	0.186	0.000^***^

*SC, Stress Control; IPE, Influence of Performance Evaluation; M, Motivation; MSK, Mental Skills; TCOH, Team Cohesion. ^†^p < 0.10; ^∗∗^p < 0.01; ^∗∗∗^p < 0.001.*

**FIGURE 1:**
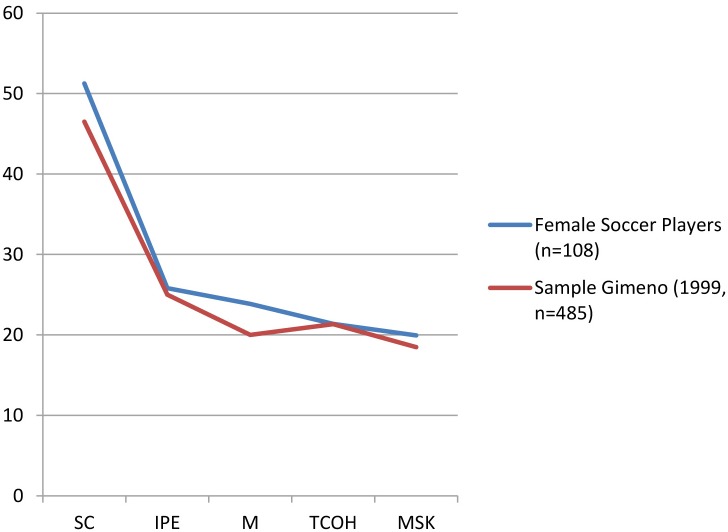
Comparative graph between the average scores of a general sample of athletes (Gimeno, 1999), and the scores of female soccer players in the present study.

Figure 1 shows the scores obtained by the players in each of the factors of the CPRD, compared with and the average scores of a general sample of athletes in the study from Gimeno (1999) with the same tool. It is noteworthy that in all the factors, the scores coming from the group of female soccer players are higher than to those from the general sample, highlighting the Motivation factor (*c* = 85, M = 23.86, *SD* = 4.04), Stress Control (*c* = 65; *M* = 51.26, *SD* = 11.15), and Team Cohesion (*c* = 65, *M* = 19.94, *SD* = 2.85), while the Influence of the Performance Evaluation (*c* = 55, *M* = 25.83, *SD* = 6.70) and Mental Ability (c = 55, *M* = 21.37, *SD* = 4.09) values are on the average.

Table 2 shows the descriptive values from the players’ self-evaluation of the performance in match 1. The answer rate was 81.5% (*n* = 88), while it was 82.4% for the second match (*n* = 89). When observing the coaches evaluations, it was initially of 83.3% (*n* = 90) in the first game, and of 84.3% (*n* = 91) in the second one. Of these last two evaluation, only 66.67% (*n* = 72) and 66.76% (*n* = 73) of valid cases have been finally considered, due to the elimination of 18 evaluations of match 1 and 18 from the match 2 due to observed answers biases (extreme scores given without discrimination of variability in scores).

**Table 2:** Descriptive values and means differences between players’ and coaches’ perceptions of performance in the two games (Wilcoxon W).

	*N*	*M*	*SD*	Minimum	Maximum	Average rank	Total ranks	*Z*	*p*
PP-1	88	7.182	1.2158	4.0	9.5	30.34	1153.00	-3.006	0.000^***^
PP-2	89	7.006	1.6140	2.0	9.5	20.75	332.00		
N valid	87								
CP-1	72	7.347	1.4551	0.0	9.0	29.08	523.50	-2.295	0.022^*^
CP-2	73	7.795	1.6726	4.0	10.0	28.22	1072.50		
N valid	71								

*PP, Players’ perceptions; CP, Coaches’ perceptions. ^∗^p < 0.05; ^∗∗∗^p < 0.001.*

We may observe also that the players’ self-evaluations are very similar between the two games, although this short difference is significant in favor of the first match (*z* = -3.606, *p* < 0.001). Regarding the evaluations made by the coaches, they are somewhat higher than those made by the athletes, finding significant differences between the performance evaluations made between the first and second match (*z* = -2.295, *p* < 0.05). Unlike athletes, coaches give a better rating to the performance obtained in the 2nd game than the one from the first game.

Table 3 shows the descriptive values and the differences of means between the under-16 and under-18 categories.

**Table 3:** Descriptive values and analysis of means differences of the two subgroups (*n* = 54 each group).

		*M*	*SD*	Average rank	Total ranks	W Wilcoxon	*Z*	Sig.
SC	Under-16	52.22	12.04969	56.97	3076.50	2809.500	-0.821	0.412
	Under-18	50.30	10.20660	52.03	2809.50			
IPE	Under-16	26.65	6.92591	58.51	3159.50	2726.500	-1.332	0.183
	Under-18	25.02	6.42663	50.49	2726.50			
M	Under-16	24.52	4.00820	59.77	3227.50	2658.500	-1.756	0.079^†^
	Under-18	23.20	4.00179	49.23	2658.50			
MSK	Under-16	21.74	3.59866	57.53	3106.50	2779.500	-1.008	0.313
	Under-18	21.00	4.53914	51.47	2779.50			
TCOH	Under-16	20.37	2.43633	60.38	3260.50	2625.500	-1.975	0.048^*^
	Under-18	19.50	3.16675	48.62	2625.50			

*SC, Stress Control; IPE, Influence of Performance Evaluation; M, Motivation; MSK, Mental Skills; TCOH, Team Cohesion. ^†^p < 0.10; ^∗^p < 0.05.*

The under-16 category players obtain higher scores than the players of the under-18 category in all the factors, although only statistically significant differences appears in Team Cohesion (*p* < 0.05.), and a tendency toward significance in the Motivation factor (*p* < 0.05).

Table 4 shows the players’ performance self-evaluations, the coaches’ evaluations and the scores obtained in the five factors of the CPRD, considering independently the under-16 category and the under-18 category.

**Table 4:** Correlation values regarding the under-16 category (*n* = 29) and under-18 (*n* = 44) using the Spearman’s Rho statistic.

Under-16					Under-18				
	PP-1	PP-2	PE-1	PE-2	PP-1	PP-2	PE-1	PE-2
PP-1	1.000				1.000			
PP-2	0.286	1.000			0.702***	1.000		
	0.141				0.000			
PE-1	0.126	0.057	1.000		0.405^*^	0.215	1.000	
	0.516	0.774			0.014	0.207		
PE-2	-0.037	0.118	0.500^**^	1.000	0.351^*^	0.445^**^	0.360^*^	1.000
	0.850	0.535	0.007		0.036	0.007	0.018	
SC	-0.126	0.231	-0.278	-0.288	0.142	0.242	-0.003	-0.120
	0.514	0.220	0.144	0.123	0.359	0.113	0.987	0.445
IPE	-0.303	0.254	-0.244	-0.086	-0.108	-0.007	-0.081	-0.237
	0.110	0.176	0.202	0.650	0.487	0.966	0.605	0.127
M	0.253	0.125	-0.255	-0.203	-0.040	-0.006	0.264^†^	-0.162
	0.185	0.510	0.182	0.283	0.798	0.971	0.087	0.299
MSK	0.331^†^	0.026	0.008	-0.154	-0.173	-0.052	0.038	-0.104
	0.079	0.891	0.969	0.416	0.261	0.738	0.809	0.506
TCOH	0.003	-0.101	0.109	-0.097	-0.479***	-0.402^**^	-0.101	-0.273^†^
	0.989	0.595	0.574	0.611	0.001	0.007	0.518	0.076

*PP, Players’ perceptions; CP, Coaches’ perceptions; SC, Stress Control; IPE, Influence of Performance Evaluation; M, Motivation; MSK, Mental Skills; TCOH, Team Cohesion. ^†^p < 0.10; ^∗^p < 0.05; ^∗∗^p < 0.01;^∗∗∗^p < 0.001.*

In the under-16 category, only correlations are found between the two coaches’ evaluations (match 1 and match 2; rho = 0.500, *p* < 0.01), and a tendency to statistical significance (*p* < 0.10) between the perception of the players in match 1 and the Mental Ability factor. On the contrary, in the under-18 category multiple results with statistical significance appears. There are important correlations between the perception of the players’ performance in match 1 and match 2 (rho = 0.702, *p* < 0.001). Also, significant correlations –with medium values- were found between the player’s perception of their performance in game 1 and the coaches’ perception in that same game (rho = 0.405; *p* < 0.05), obtaining values significantly higher when considering the second match (rho = 0.445; *p* < 0.01).

Regarding to the CPRD factors, the Team Cohesion obtains significant inverse correlations of medium values when correlated with the players’ perception in matches 1 (rho = -0.479; *p* < 0.001) and 2 (rho = -0.402; *p* < 0.01). The same factor have a significant negative tendency (rho = -0.273; *p* < 0.10) correlated with coaches’ evaluation in match 2. Added to this, there is a positive correlation with tendency to the significance between the Motivation factor and the coach’s evaluation in the first game (rho = -0.264; *p* < 0.10).

## Discussion

Studying the psychological profile of female soccer players, as was indicated by Rutkowska and Bergier (2015), is extremely important, and also can provide many interesting ideas about the specific nature of women’s youth soccer.

It can also show where improvements can be made in sports training programs, specifically in mental training, especially in those players who will soon become part of professional football (MacNamara and Collins, 2017). In this vein, the objectives of this study have been to describe the psychological characteristics of young female soccer players, determine the differences between the under-16 and under-18 categories, showing their relationship and congruence with the perception of performance, from both the players and coaches.

The results show that the scores of the young soccer players in the CPRD are higher than those of the general sample of athletes, especially in the scales of M, SC, and TCOH. Specifically in M they have a score that can be considered as quite high, that is, the players in the sample studied seem to have an adequate motivational state to meet the demanding requirements of sports practice and the competition, which coincides with other studies on female soccer players (Muñoz et al., 2018), in which the basic motivation found was intrinsical, related to the game itself and by being part of a team, although these features have fluctuations depending on the competition level (Domínguez-Escribano et al., 2017).

Both in the SC and in TCOH subscales the scores obtained are moderately high, which in principle seems to indicate that the players tend to control well the potential stress inherent to the competition, and that they have a good disposition to work with the team. Regarding the scores obtained in IPE and MSK, the results indicate that they are on the average, which in the first case indicates that the players can be negatively affected by the evaluation that other people (or indeed themselves) make of their performance, and in the second case indicates that the players do not have full consolidated psychological skills, needed for to perform optimally. This last factor affects the need to propose psychological intervention programs tailored to the specific needs and characteristics of athletes, depending on the competition level, gender, etc. In the same vein, Slimani et al. (2016) performed a systematic review finding some very interesting data showing the differences in cognitive/psychological training interventions, and their effectiveness, depending on whether they are directed toward practices or competition, age, competition level and the players’ position.

Considering that the players have a similar level of performance in their respective categories, it is clear they show some overestimation of their skills (mainly perceived in the under-16 category), perhaps due to a fewer competitive experience, a lower self-awareness of psychological limitations, and less exposure to experiences related to the performance in competition demands. Also, from an evolutionary perspective, players of 14 and 15 years old are in the middle of the adolescence, where variables such as self-concept and self-knowledge are under construction, and therefore, may have a minor adjustment of their psychological skills to deal with situations of stress and promote self-confidence (Sarkar and Fletcher, 2014; González-Campos et al., 2015; Gómez-Espejo et al., 2017).

On the other hand, with respect to the second objective, the results indicate that the under-16 players obtain higher scores than the under-18 players in all the factors, showing statistically significant differences in TCOH and a tendency to statistical significance in M. In any case, the most relevant differences are in the TCOH scale, where perhaps the younger players have a better disposition to work inside the team, while the U18 players have other aspects of personal competence could work against the teamwork; and on the M scale, which in this case may be better understandable due to other major competitive factors, such as being near the end of the youth stage in the case of the U-18, when some of the players could to be thinking about alternatives outside the practice of competitive football, or that the perceived pressure is inadequately managed, generating anxiety and worry. These data are in accord with what other found in other studies (Pan and Zhu, 2003), where at a higher level of psychological competitiveness, lower is the level of worry and anxiety and better the performance.

Finally, regarding the third objective, and taking into account the whole sample, the results indicate that the players’ self-evaluations are very similar between match 1 and match 2, although this difference is significant in favor of match 1. In other words, they perceived that they have played the first match better than the second. Interestingly, the evaluations made by the coaches are somewhat higher than those made by the players themselves, finding significant differences between the assessments made between the match 1 and 2; unlike the players, the coaches give a better rating to the performance obtained in match 2. In this sense, some studies (Møllerløkken et al., 2017) have also found differences between the perception of players and coaches regarding the motivational climate, meaning that players of both sexes perceived that the motivational climate was more oriented toward the performance and less toward the domain compared to coaches. In both cases, these findings may improve our understanding of the coach-player relationship, and may be important in understanding the players’ motivational style. Indeed, Reid and Crust (2012) examined the perceptions of elite soccer players and coaches about the psychological drive and how it relates to objective performance, finding that teammates and spectators can have a greater impact on the momentum in female football than with the male soccer.

When analyzing the data by categories, the results indicate that in the under-16 category, only correlations are found between the coaches’ evaluation of first and second matches. On the other hand, in the under-18 category there are quite relevant correlations between the perception of the player’s performance between the two matches; between the players’ and coach performance perception match 1 and the perception, indeed reaching higher significant values when considering the second game. In this last case, the under-18 players’ great experience could explain, in part, the correlation of their performance perception with that of their coach, since in the under-16 category that lack of experience could play against a more realistic self-evaluation, differing more with the most experienced coach evaluation.

Furthermore, this fact supports the fact that the TCOH factor obtains significant inverse correlations -of medium values- when is correlated with the player’s self-perception both in the first and second matches, and when at the same time obtain a significant negative tendency with the coach’s evaluation in the second game. In short, these results indicate that the under-18 players have performance perceptions more adjusted to the expert criterion (from the coaches) than the under-16; the higher experience in the under-18 players’ sport practice could explain these differences between these both groups.

Finally, the CPRD questionnaire seems to be a very appropriate instrument to describe the relevant psychological characteristics related to sports performance, and to provide very valuable information, both from a group and individual point of view, serving as a basis for implementing training programs and specific psychological training for the team or individual level. As indicated by Olmedilla et al. (2017a) the knowledge of the psychological characteristics of young athletes can be very valuable so that, together with the physical and anthropometric indicators, they allow their coaches to individualize the training processes and thus optimize them.

The present study provides fundamentally some knowledge about the psychological variables of women’s competitive soccer, showing psychological differences in two youth sports categories where the player’s experience and age may have great relevance (Ruiz, 2005), as well as the relationships between the load of practice and/or competitions regarding players’ mood, psychological well-being and physiological well-being (Lowe, 2017; McFadden et al., 2017; Tharawadeepimuk and Wongsawat, 2017), especially in view of the possibility of evaluating and intervening on a psychological level in women’s football teams (Olmedilla et al., 2018b).

The results of this study can help to establish the differences between different constellations of psychological characteristics related with performance levels and their relationship with the subjective perception of performance. This knowledge can be used by sports professionals: coaches, psychologists, physical educators, etc., to help athletes achieve their maximum performance, and to implement effective intervention programs for sports performance (MacNamara et al., 2010; Bennett and Maynard, 2016; Gledhill et al., 2017); personal growth and athletes’ dual-career (Llames and García-Dantas, 2017), or even athletes’ injury prevention (Olmedilla et al., 2017b).

### Limitations and Future Research Directions

In the present study, the participating regional teams have been analyzed in the final phase of the national 11 women’s soccer championship in the under-16 and under-18 categories, with 3 teams in each category. The non-randomized selection of the participants, the specific context of the competition and the small sample size of the current research imply that the results obtained cannot be generalized, and that they cannot be compared with other performance levels.

On the other hand, it would be interesting to collect data from a greater number of matches, and perhaps to include practices, in order to determine the differences in the player’s and coaches’ perception of performance between the two situations. In addition, the coaches’ evaluation carried out is individual and not peer-reviewed, and in subsequent studies it should be good to carry out inter-judge analyzes obtaining a more reliable expert criterion regarding the data analysis.

Likewise, there is a great dispersion in the number of hours and days of practice in the players assessed, what is a representation of the reality of female soccer in Spain; so it could be interesting to study more homogeneous groups in future approaches. Regarding future research developments, should be interesting made up studies where specific changes of psychological skills in different female sports teams may be analyzed. Likewise, it would be convenient to establish comparisons with male athletes, aiming to tailoring accurately eventual psychological interventions addressed to each epidemiological segment (such as gender, sport type, athletes’ age, and level of performance).

## Conclusion

The scores of the young soccer players in the CPRD are higher than those of the general sample of athletes, especially in the scales of M, SC, and TCOH.

The under-16 category players’ obtained higher scores than the under-18 category players’ in all factors, showing statistically significant differences in TCOH, and a tendency to statistical significance in M.

The players’ self-perception of their performance is very similar between match 1 and match 2, although the small difference is significant in favor of match 1.

The players’ performance made by their coaches is somewhat higher than those made by the players themselves.

## Ethics Statement

This study was carried out in accordance with the recommendations of the Declaration of Helsinki. The protocol was approved by the Comité de Ética de la Universidad de Murcia (reference: UM 1551/2017). All subjects gave written informed consent in accordance with the Declaration of Helsinki.

## Author Contributions

AO and AG-M contributed with the conception and design of the study. AO and RR-B organized the database. RR-B and FP performed the statistical analysis. AO wrote the first draft of the manuscript. FP, AG-M, RR-B, and FR-P wrote the sections of the manuscript. FR-P was in charge of the formal aspects of the work. All authors contributed to the revision of the manuscript, and read and approved the presented version.

## Conflict of Interest Statement

The authors declare that the research was conducted in the absence of any commercial or financial relationships that could be construed as a potential conflict of interest.
